# Effect of synchronicity of amino acid supply on the synthesis of protein in C2C12 myotubes cultured *in vitro*

**DOI:** 10.3389/fvets.2024.1423604

**Published:** 2024-10-29

**Authors:** Qiyu Zhang, Mengmeng Mi, Tianjiao E, Xin Fu, Nan Bao, Li Pan, Yuan Zhao, Guixin Qin

**Affiliations:** Key Laboratory of Animal Production, Product Quality and Security, Ministry of Education, Jilin Provincial Key Laboratory of Animal Nutrition and Feed Science, College of Animal Science and Technology, Jilin Agricultural University, Changchun, China

**Keywords:** nutrition, C2C12 myotubes, amino acid supply, synchronicity, protein synthesis

## Abstract

Previous studies inferred that the synthesis rate/efficiency of protein in body tissue is probably affected by synchronicity of different amino acid (AA) supply in its metabolic pool. In order to further observe the influence of synchronicity of AA supply on the synthesis of protein in cell level, a cell culture experiment *in vitro* was conducted with C2C12 myotubes. C2C12 myotubes were cultured for 24 h, meanwhile the culture medium was replaced for each 8 h. Those myotubes were subjected to 3 treatments (1 for controlled and 2 for tested), control myotubes were cultured with same normal complete medium within the whole 24 h, and the 2 tested myotubes were cultured with asynchronous amino acid supply medium in which the levels of different AAs (Lysine, threonine, methionine, leucine, valine and glutamic acid) either increased and then decreased or decreased and then increased, at different replaced medium time point (at 0, 8, and 16 h). However, during the whole experiment period all the 3 treated myotubes received same amount of each AA. The sample of the myotubes were used for myotube morphology, protein, AA, and proteomic analysis. The results showed that asynchronous AA nutrition affect the synthesis and degradation of myotube proteins, and the AAAS in the medium increase, thus decreasing the synthesis rate of myotube proteins (*p* < 0.05) and decreasing the diameter of myotubes (*p* < 0.05). The process of reduced protein synthesis affects the PI3K-AKT and FoxO signaling pathway by downregulating the levels of IRS1 and EGFR, and the degradation amplitude is greater than the synthesis amplitude. Therefore, this study further revealed the effect of the asynchronous supply of amino acids on myotube protein synthesis and the underlying mechanism and provided a theoretical reference for the precision of nutrition to animals.

## Graphical abstract

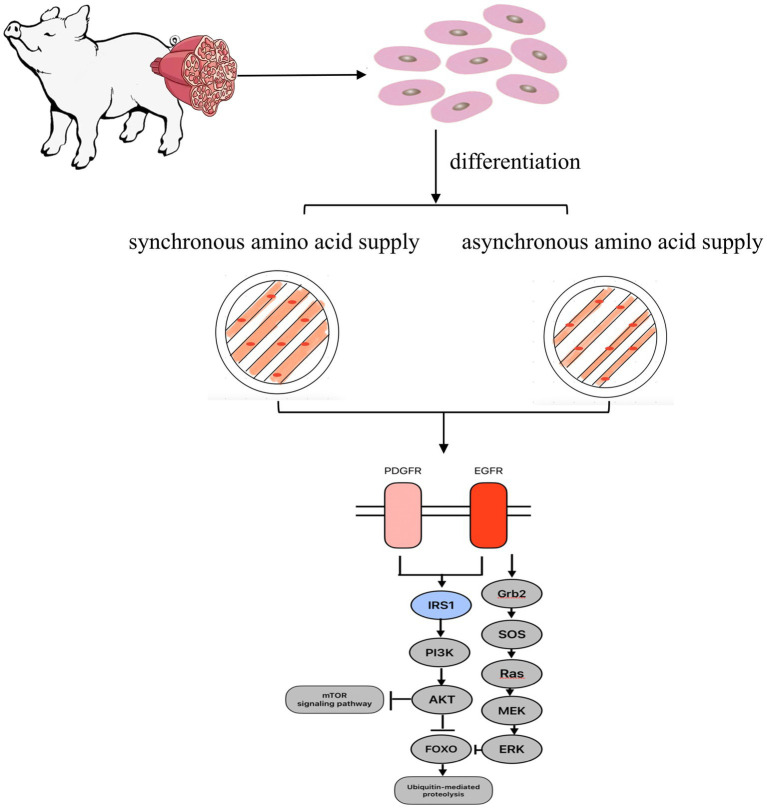

## 1 Background

According to the previous animal nutrition theory, it is generally believed that whether the nutrients in the diet can meet the nutritional needs of animal life activities and production mainly depended on the type, quantity and proportion of effective nutrients provided by the diet (1). In terms of protein and amino acid nutrition of pigs, both the expression of nutritional value in feed and the expression of nutritional requirements of pigs are measured by the digestible quantity at the end of the ileum (2, 3). However, many domestic and foreign studies have proved that balanced diets formulated with different protein sources and the same digestible amino acids in the end of the ileum cannot obtain stable results, and the growth performance and nitrogen deposition effect of pigs are very different (4, 5). Further studies have proved that nitrogen deposition efficiency is likely to be related to the equilibrium of amino acid digestion time and the synchronization of different kinds of amino acids in the diet (6, 7).

The main synthesis site of amino acids for protein deposition is in skeletal muscle (8), which accounts for about 40% of the total body mass (9). The regulation of skeletal muscle mass depends on the dynamic balance between amino acid protein synthesis and degradation (10, 11), so enhancing the efficiency of skeletal muscle protein deposition is crucial for livestock meat production (8). Some studies have shown that the protein synthesis rate of various tissues and organs of piglets was detected when feeding unbalanced amino acid diets, and the results showed that the growth of piglets was limited by the decrease of the protein synthesis rate of skeletal muscle (12, 13). Therefore, observing the protein synthesis of muscle cells is the main target to accurately reflect the protein synthesis status of the body. If the synthesis of proteins in the body is influenced by the pattern of amino acid supply, then the synchrony and temporal balance of amino acid supply may affect protein synthesis in muscle cells.

Many studies exploring protein nutrition in skeletal muscle have used the C2C12 cell line as an *in vitro* model (14, 15). Therefore, this study verified and revealed the regulatory mechanism of amino acid release synchronization affecting protein synthesis through *in vitro*. In the control group, the normal medium was used as synchronous amino acid supply mode, and the C2C12 myotube was cultured by customized medium in the asynchronous amino acid supply group.

## 2 Materials and methods

### 2.1 Cell culture and differentiation

Mouse myoblast C2C12 cells were purchased from Shanghai EK-Bioscience Biotechnology Co., Ltd., inoculated in cell culture bottles and cultured in DMEM/high glucose medium (icell, Shanghai, China). The medium was supplemented with 1% penicillin–streptomycin (cytiva, SV30010, Utah, America) and 10% fetal bovine serum (Clark, FB25015, Australian Origin) at 37°C in a humidified incubator (HERA CELL 240i; Thermo, USA) with 5% CO_2_. After the cells had proliferated to 80%, the medium was changed to differentiation medium [DMEM containing 2% horse serum (Cytiva, SH30074, USA) and 1% penicillin–streptomycin solution] for 5 days. The medium was changed every two days after two washes with PBS (cytiva, SH30256.01, USA) to promote maturation of mature multinucleated myotubes.

### 2.2 Treatment scheme

Fully differentiated myotubes were treated with two media with different AA concentrations for 24 h, and the medium was changed every 8 h. The synchronized amino acid supply (SAAS) was cultured with normal medium, and the asynchronous amino acid supply (AAAS) was cultured with customized medium. The amount of each addition was 2 mL. The order of medium addition was 1–2-3 in the first group and 2–1-3 in the second group for the AAAS pattern. According to the feature that the amino acid concentration in portal vein blood of pigs increased first and then decreased after eating different protein source diets with different free amino acid concentration *in vitro* (16), the cell medium supply formula with unsynchronized amino acid supply was designed. The AAs selected for adjustment were mainly considered as follows: one is the limiting AA related to muscle protein synthesis (17), and the other is the branched-chain AA, which is the main source of nitrogen metabolism energy in skeletal muscle (18). To prepare the medium with the asynchronous amino acid supply, the sequence in which the concentration of lysine, threonine and methionine rose first and then fell was adjusted, and the sequence in which leucine and valine fell first and then rose was also adjusted. The concentration of glutamate was used to balance the total molar number of AA in each period, which was consistent with that of the control group. The specific formula is shown in Table 1.

**Table 1:** Test medium formulation (concentration, mg/L).

Component description	SAAS^1^	AAAS^2^		
		1	2	3
Inorganic salts				
Calcium Chloride	200	200	200	200
Ferric Nitrate-9H_2_O	0.1	0.1	0.1	0.1
Potassium Chloride	400	400	400	400
Magnesium Sulfate	97.67	97.67	97.67	97.67
Sodium Chloride	6,400	6,400	6,400	6,400
Sodium Phosphate monobasic H_2_O	125	125	125	125
Amino acids				
Glycine	30	30	30	30
L-Arginine-HCl	84	84	84	84
L-Cystine-2HCl	63	63	63	63
L-Glutamine	584	498.25	701.5	552.25
L-Histidine-HCl-H_2_O	42	42	42	42
L-Isoleucine	105	105	105	105
L-Leucine	105	78.75	78.75	157.5
L-Lysine-HCl	146	219	109.5	109.5
L-Methionine	30	45	22.5	22.5
L-Phenylalanine	66	66	66	66
L-Serine	42	42	42	42
L-Threonine	95	142.5	71.25	71.25
L-Tryptophan	16	16	16	16
L-Tyrosine-2Na-2H_2_O	104	104	104	104
L-Valine	94	70.5	70.5	141
Total number of moles of amino acids	1,606	1,606	1,606	1,606
Vitamins				
Calcium Chloride (CaCl_2_) (anhyd.)	200	200	200	200
Ferric Nitrate (Fe(NO3)3”9H_2_O)	0.1	0.1	0.1	0.1
Magnesium Sulfate (MgSO_4_) (anhyd.)	97.67	97.67	97.67	97.67
Potassium Chloride (KCl)	400	400	400	400
Sodium Bicarbonate (NaHCO_3_)	3,700	3,700	3,700	3,700
Sodium Chloride (NaCl)	6,400	6,400	6,400	6,400
Sodium Phosphate monobasic (NaH_2_PO_4_-H_2_O)	125	125	125	125
Other				
D-Glucose (Dextrose)	4,500	4,500	4,500	4,500
HEPES	0	0	0	0
Phenol Red	15	15	15	15
Sodium Pyruvate	110	110	110	110

^1SAAS, medium with synchronous amino acid supply. 2AAAS, medium with asynchronous amino acid supply.^

### 2.3 Determination of myotube protein

After 5 days of myocyte differentiation and the formation of mature myotubes, the untreated myotubes were set as blanks. Myotubes were collected from the SAAS group and AAAS group after treatment for 24 h. Cells were lysed with a total protein extraction kit (Bestbio, BB-3101, Shanghai China) for 30 min on ice. Whole cell lysates were centrifuged at 12,000 × g for 15 min at 4°C, and the supernatant was transferred to a new tube. The protein concentration of each sample was quantified using the BCA Protein Assay kit (Shanghai Epyzime Biotechnology Co., Ltd., ZJ102L, Shanghai, China). The protein content of the SAAS group and AAAS groups was obtained by subtracting the blank space.

### 2.4 Assay of urea content in the supernatant of myotubes

The mature myotubes were treated for 0, 8, 16, and 24 h, and the culture medium supernatant was collected. The urea content in the samples at each time point was determined by a urea nitrogen content assay kit (Boxbio, AKM002M, Beijing China), and the sum was used as the total urea content for statistical analysis. According to the results of protein determination, the AAAS1 group with lower protein of myotubes and higher urea nitrogen of medium was selected to detect the following items.

### 2.5 Determination of AA in myotube supernatant

During the treatment period, samples were collected every eight hours, and the initial myotubes medium supernatant was analyzed as follows.

Step 1: Sample pretreatment was performed as described by Dai et al. (19). The content of free AA in serum was determined by high-performance liquid chromatography (HPLC). Sample pretreatment: Samples were removed from −80°C, and a 200 *μ*L aliquot of the samples were placed into a 1.5 mL centrifuge tube after melting. Then, 200 μ L of 1.5 mol/L HCIO_4_ was added, shaken, mixed and left to stand at 4°C for 30 min. To adjust the pH of the solution, 200 *μ* L of 2 mol/L K_2_CO_3_ was added, shaken and mixed well. The samples were centrifuged at 10000 r/min for 2 min, 100 μL of supernatant was placed into a 1.5 mL centrifuge tube, and 100 μL of 1.2% benzoic acid and 1,400 μ L of ultrapure water were added. The solutions were mixed and transferred to a UPLC automatic sampling bottle for analysis.

Step 2: Sample determination A Waters ACQUITY UPLC FLR system (Waters, Milford, MA, USA) and an AcQUITY UPLC column (2.1 mm x 100 mm, P/N:186002352) were used with the following parameters. Mobile phase A (0.1 M sodium acetate, pH 7.2), 27.3 g sodium acetate trihydrate was added to 1.6 L H_2_O in a glass bottle, followed by sequential addition of 96 μL of 6 M HCl, 180 mL methanol, and 10 mL tetrahydrofuran. After the mixture was evenly mixed, H_2_O was added, and the final volume of the solution was 2 L. Mobile Phase B: 100% methanol, brown glass bottle. Flow rate, 0.3 mL/min; injection volume, 1 μL; column temperature, 35°C; sample temperature, 5°C; detection wavelength, 450 nm; collection rate, 20 points/s; time constant, 0.1 s; and run time, 19 min.

By calculating the percentage of the remaining amino acid content of each amino acid in the initial medium every 8 h, the synchronization of amino acid absorption at each time point was obtained. The absorption kinetics curves of a single AA were plotted using Origin 9.1 (USA) software. The synchronization degree of AA was quantified according to a method by Wang et al. (6). The standard deviation of AA release (%) of monomer at each time point was calculated, which was used as the index of AA release synchronously. The synchronization index (SI) of each medium is the sum of the synchronization index of each time point. The lower the SI is, the better the synchronization.

### 2.6 Nonradioactive measurements of protein synthesis with SUnSET

Protein synthesis was measured using translational surface sensing technology (SUnSET) (20). At the bottom dose level, puromycin binds to the newly synthesized protein, directly reflecting the translation rate of mRNA *in vitro* (21, 22). C2C12 cells were cultured as described above and incubated with 1 μg/mL puromycin (23) for 30 min. After the myotubes were collected in ice-cold cell lysate, the incorporation changes of puromycin was detected by western blotting (24).

### 2.7 Immunofluorescence staining

The test method was based on the Caldow method (25). The cultures were removed by suction, and the cells were washed (2 × 5 min) with phosphate-buffered saline (PBS) and fixed in 4% paraformaldehyde (Beijing Dingguo Changsheng Biotechnology Co. LTD, AR-0211, Beijing, China) for 15 min. The cells were then washed with PBS (3 × 5 min), infiltrated with PBS containing 0.1% Triton X-100 (Coolaber, DZSL1466-100, Beijing, China) for 20 min, washed with PBS (3 × 5 min) and incubated for 2 h in PBS containing 3% bovine V albumin (Biotopped, A6020, Beijing, China). Then, the cells were then washed with PBS (3 × 5 min), incubated with MYH primary antibody (1:300, sc-376157, Santa Cruz, Dallas TX, USA) overnight at 4°C, washed with PBS (3 × 5 min) and incubated with YF488 goat anti-rabbit secondary antibody (1:500, Y6105L, UElandy, Suzhou, China) for 2 h at room temperature in the dark. The cells were then washed with PBS (3 × 5 min) and incubated with 1× DAPI staining solution (readyuse type) (SL7100, Coolaber, Beijing, China) for 5 min at room temperature in the dark to stain nuclei. Finally, the cells were washed again with PBS (3 × 5 min). Myotubes were photographed by scanning with a laser confocal microscope (Leica SP8, Germany).

### 2.8 Measurement of myotube diameter

Myotubes were photographed with a laser confocal microscope, and their diameters were measured using Leica software. The average diameter of myotubes was calculated by measuring the maximum diameter of each. Ten random culture blocks were taken for each sample, and at least 100 myotubes were counted.

### 2.9 Proteomic analysis of protein synthesis and degradation pathways in myotubes

#### 2.9.1 Protein extraction and peptide enzymatic hydrolysis

An appropriate amount of SDT (4% (w/v) SDS, 100 mM Tris/HCl, pH 7.6) lysate was added to all samples for protein extraction, followed by protein quantification by BCA assay. Twenty micrograms of protein from each sample was added to an appropriate amount of 5X loading buffer, subjected to SDS–PAGE (4–20% preformed gradient gel, constant pressure 180 V, 45 min) in a boiling water bath for 5 min, and stained with Coomassie Brilliant blue R-250. The expression level of protein in the samples was quantified by Shanghai Personal Biotechnology Co., Ltd. (Shanghai, China). An appropriate amount of protein from each sample was trypsinized by the filter-aided proteome preparation (FASP) method (26), and the peptide was desalted by a C18 cartridge. The peptide was lyophilized and redissolved by adding 40 μL 0.1% formic acid solution. Peptide quantification was performed (OD280).

#### 2.9.2 LC–MS/MS data acquisition

Each sample was separated using an HPLC liquid phase system NanoElute with a nanolitre flow rate. Buffer A was 0.1% formic acid aqueous solution, and buffer B was 0.1% formic acid acetonitrile aqueous solution (acetonitrile 99.9%). The column was equilibrated with 95% liquid A, and samples were separated on a C18 reversed-phase analytical column (Thermo Scientific EASY column, 25 cm, ID 75 μm, 1.9 μm) at a flow rate of 300 nL/min.

Samples were separated by chromatography and analyzed by mass spectrometry using a timsTOF Pro mass spectrometer. The ion source voltage was set at 1.5 kV, MS and MSMS were detected and analyzed by TOF, and the scanning range of the MS spectrum was set at 100–1700 m/z. The data acquisition mode was parallel cumulative serial fragmentation (PASEF) mode. The specific data acquisition parameters were as follows: ion mobility (1/K0) was 0.6–1.6 *Vs*/cm2, and one primary mass spectrum corresponded to 10 secondary spectra of PASEF mode. The dynamic exclusion time for tandem MS scanning was set to 24 s to avoid repeated scans of parent ions.

#### 2.9.3 Protein identification and quantitative analysis

The original mass spectrometry analysis data are shown in file.d. MaxQuant software (version 1.6.14) (27) was used for library identification and quantitative analysis. Statistical analysis of proteomic results. In different proteins, the expression ratio (fold change, FC) >2.0 (increase greater than 2.0 times or decrease less than 0.5 times) and *p* < 0.05 (T test or other) were taken as the standard.

#### 2.9.4 Bioinformatics and statistical analysis

First, the quantitative information of the target protein set was normalized (normalized to the interval (−1,1)). Then, the ComplexheatmapR package (RVersion3.4) was used to classify both sample and protein expression dimensions simultaneously (distance algorithm: Euclidean, connection method: Averagelinkage) and generate hierarchical clustering heatmaps.

Second, Blast2GO (version number: BLASTP 2.8.0+) was used to perform GO annotation with the target protein set. The process can be roughly summarized into the following steps: sequence alignment (Blast), GO item extraction (Mapping), GO Annotation and InterProScan supplementary annotation (Annotation Augmentation). KOBAS (version: KOBAS3.0) software was used to annotate the KEGG pathway of the target protein set. The target protein set was compared with the distribution of each GO classification (or KEGG pathway, or Domain) in the target protein set and the overall protein set by Fisher’s exact test (Fisher’s exact test), and the enrichment analysis of GO annotations or (or KEGG pathway, or Domain) annotations was performed on the target protein set. Based on IntAct^1^ or STRING,^2^ the information in the database was used to find the direct and indirect interactions between target proteins. The interaction network was generated and analyzed.

### 2.10 Western blotting

The treated myotubes were cleaved on ice with a total protein extraction kit (Bestbio, BB-3101, Shanghai China) for 30 min. The whole cell lysate was centrifuged at 12000 × g at 4°C for 15 min, and the supernatant was transferred to the new tubes. The protein concentration of each sample was quantified by BCA protein assay kit (Shanghai Epyzime Biotechnology Co., Ltd., ZJ102L, Shanghai, China).

Equal amounts of proteins from different samples were loaded onto 7.5, 10%, or 12.5% SDS (Shanghai Epyzime Biotechnology Co., Ltd., PG113, PG114, PG112, Shanghai, China) polyacrylamide gels and separated by electrophoresis. Proteins were transferred to PVDF membranes and subsequently blocked with 5% bovine V albumin for 1 h at room temperature. Membranes were incubated with different primary antibodies against p-4E-BP1 (1:100, Santa Cruz, sc-293124, Dallas, USA), p-mTOR (1:1000, Abcam, AP0115, Cambridge, UK), IRS1 (1:100, Santa Cruz, sc-8038, Dallas, USA), EGFR (1:100, Santa Cruz, sc-373746, Dallas, USA), PDGFR (1:100, Santa Cruz, sc-80991, Dallas, USA), p-ERK (1:100, Santa Cruz, sc-7383, Dallas, USA), p-Akt (1:500, Cell Signaling, 4,060 T, Boston, USA), FOXO1 (1:1500, GeneTex, GTX135251, Irvine California, USA), ubiquitin (1:100, Santa Cruz, sc-8017, Dallas, USA), puromycin (1:5000, Sigma, MABE343, Darmstadt, Germany), and *β*-actin (1:100000, ABclonal, AC026, Cambridge, UK). The membranes were then washed three times with Tris-buffered saline-Tween 20 (TBST) (iScience, CP17207M, Jiangsu, China) solution and incubated with the corresponding anti-mouse or anti-rabbit secondary antibody (1:5000, SA00001-2, SA00001-1, Proteintech Group, Shanghai, China) for 1 h at 37°C. Finally, the membranes were washed three times with TBST and developed with a hypersensitive ECL chemiluminescence kit (NCM Biotech, P10200, Suzhou, China) and a Western blot detection system (Kodak, USA). The intensity of the bands was quantified using ImageJ software (National Institutes of Health, USA), and the relative expression of the target protein was normalized to that of *β*-actin.

### 2.11 Statistical analysis

Proteome sequencing was performed in 4 replicates, and all other tests were performed in 3 replicates. The data were sorted through Excel. SPSS 23.0 software was used to analyze the single factor (ANOVA) significance of the data, *p* < 0.05. 0.05 was significant. Plot with GraphPad Prism 10.0.2. In addition to otherwise stated statistical results, other results are provided to facilitate visualization and to normalize the data to the relative content of the SAAS control group.

## 3 Results

### 3.1 Effect of asynchronous AA supply pattern on protein deposition in myotubes

**Figure 1:**
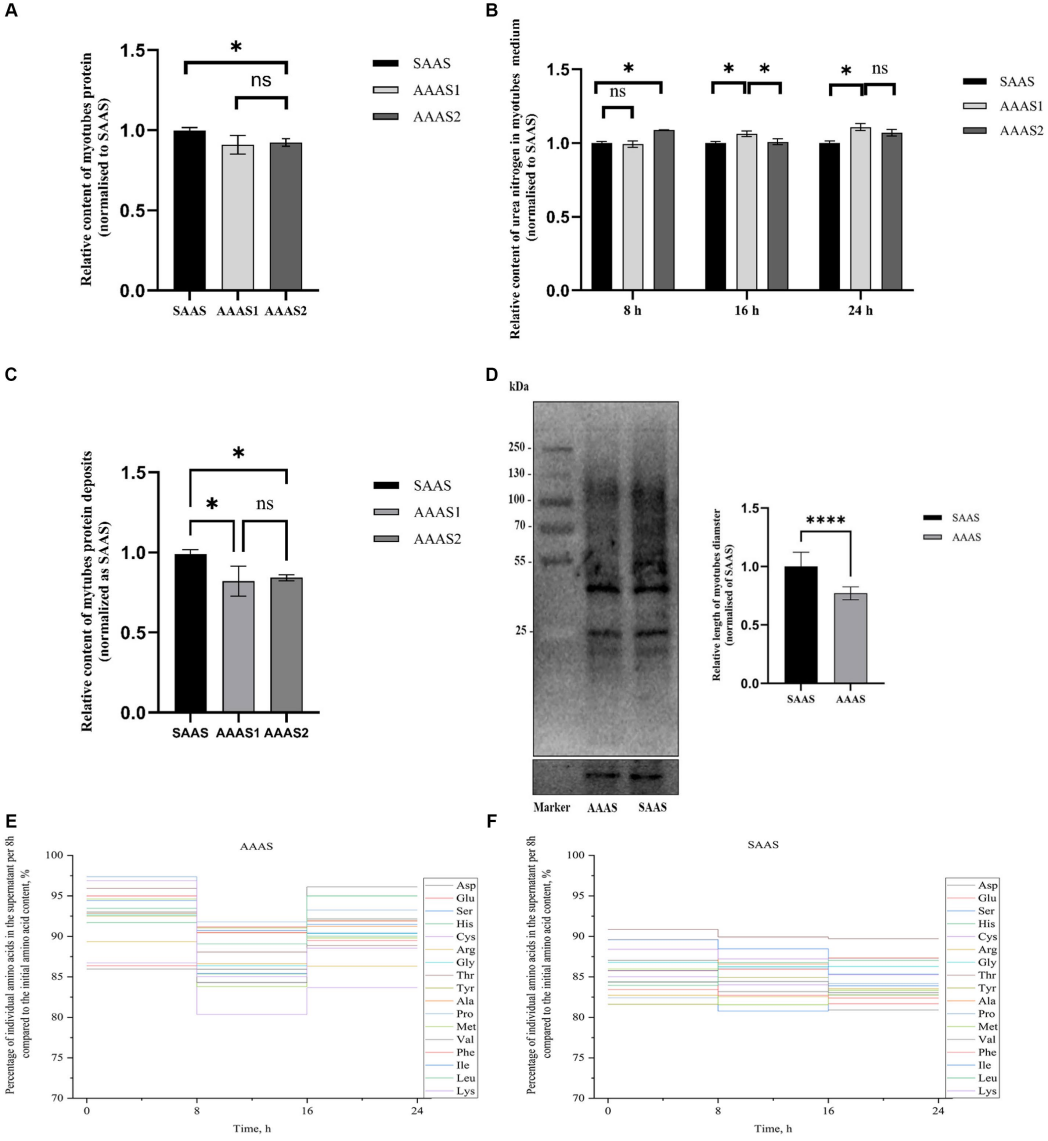
Effect of asynchronous AA supply pattern on myotube protein deposition. **(A)** The asynchronous AA supply pattern affects the protein content of myotubes. **(B)** The asynchronous AA supply pattern affects the urea nitrogen in myotubes medium. **(C)** The asynchronous AA supply pattern affects the protein deposition of myotubes. **(D)** Effect of the AA supply pattern on protein synthesis in myotubes. **(E)** Percentage of individual amino acids in the supernatant per 8 h compared to the initial AA content. **(F)** Synchronization index (SI) of AA 1 (%). AAAS, medium with asynchronous AA supply; SAAS, medium with synchronous AA supply.

The total protein content of myotubes in the SAAS group was significantly higher than that in the AAAS group when the myotubes were cultured for 24 h in the asynchronous amino acid supply pattern (*p* < 0.05, Figure 1A). However, urea nitrogen production in the SAAS group was significantly lower than that in the AAAS group (*p* < 0.05, Figure 1B). According to the results of protein deposition, AAAS1 was selected as the representative of the asynchronous amino acid supply pattern (*p* < 0.05, Figure 1C). The content of newly generated proteins in myotubes in the SAAS group was significantly higher than that in the AAAS group (*p* < 0.05, Figure 1D).

For 17 individual AAs, a vertical step curve of AA release every 8 h, which was expressed as a percentage of the final concentration, was plotted to indicate AA absorption synchronization. Compared with the medium with SAAS, the AA synchronicity of the medium with AAAS was poor in each time (Figure 1E). Moreover, the synchronization index of AA every 8 h is shown in Table 2. The proportions of AAAS and SAAS media were 9.70 and 7.53%, respectively. The SI of the AAAS medium is higher than that of the SAAS medium.

**Table 2:** Asynchronization index of AA (%).

Time points, h	AAAS	SAAS
0–8	3.49	2.69
8–16	3.26	2.46
16–24	2.96	2.37
Summation^1^	9.70	7.53

^1The summation was calculated from the sum of the SI at each time point. The lower the summation value is, the better the synchronization.^

### 3.2 Effects of AA supply pattern on C2C12 myotubes size

**Figure 2:**
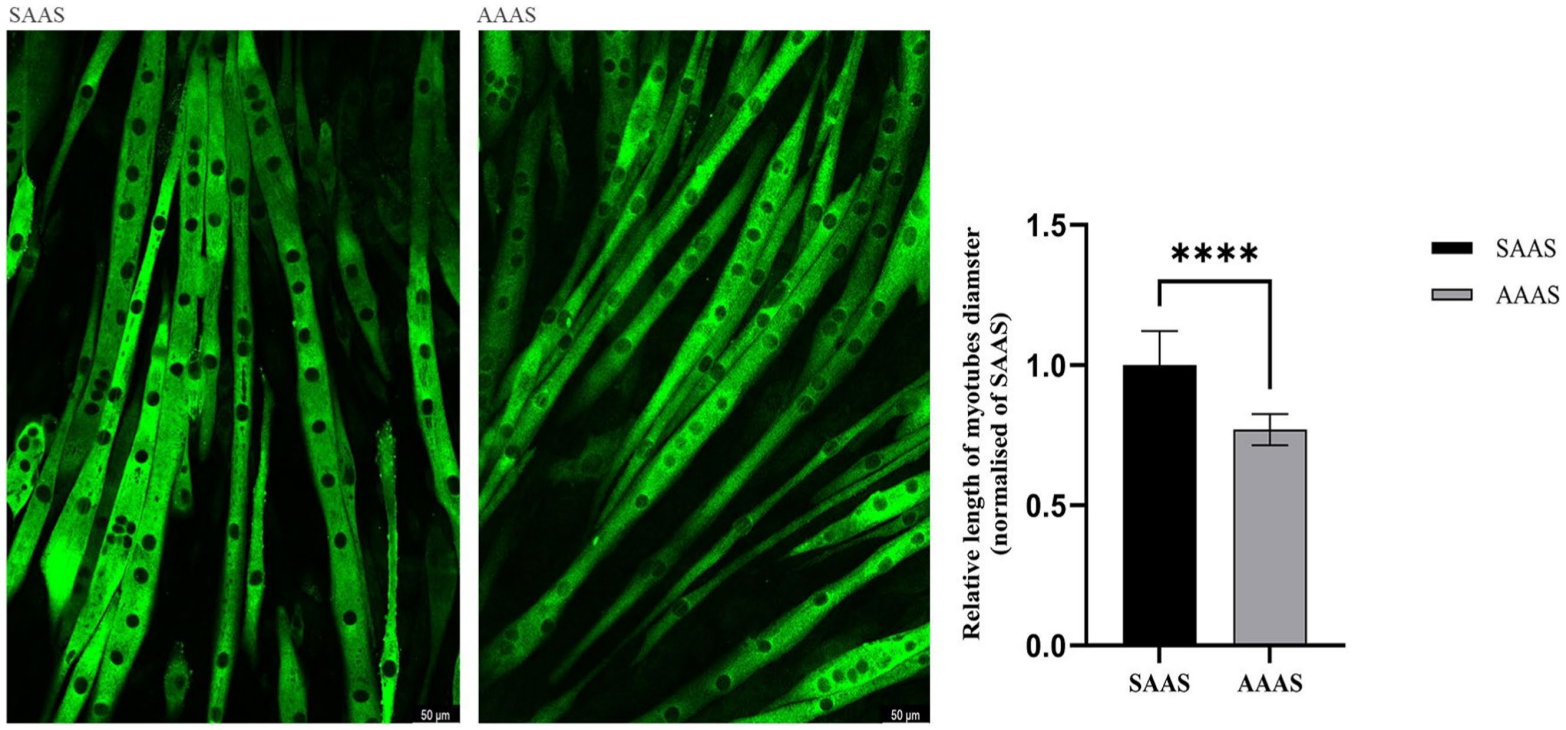
Effect of asynchronous AA supply pattern on the size of C2C12 myotubes. AAAS, medium with asynchronous AA supply; SAAS, medium with synchronous AA supply.

Unconventional amino acid supply (24 h) in DMEM resulted in slow growth of C2C12 myotubes. Compared with SAAS, the differentiation medium with AAAS presented a decrease in myotube diameter (*p* < 0.05; Figure 2).

### 3.3 Identification and quantification of myotube proteins

**Figure 3:**
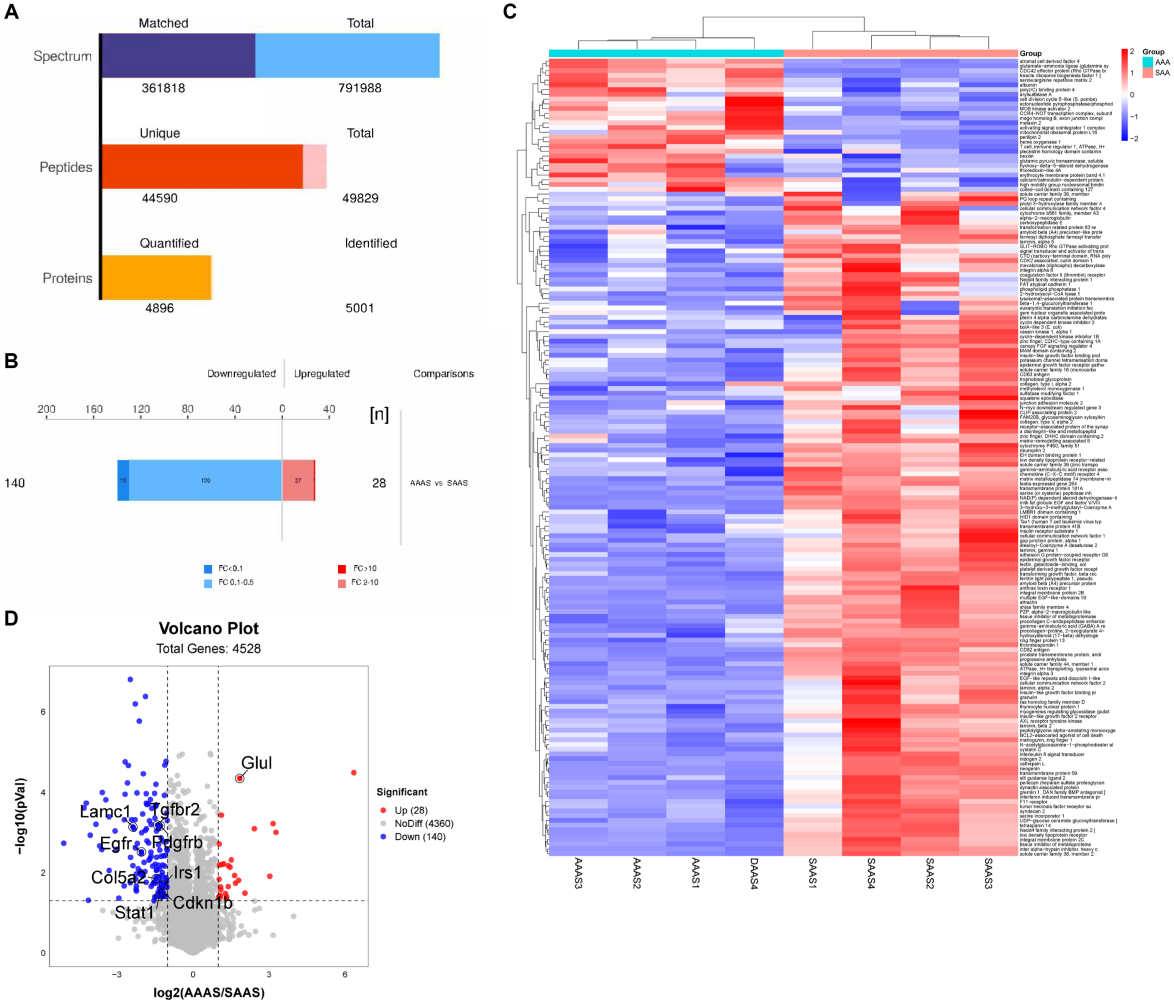
Global proteomic changes in C2C12 myotubes after AAAS and SAAS treatments. **(A)** Statistical histogram of identification and quantitative results. **(B)** Protein quantitative difference histogram. **(C)** RNA sequencing analysis of myotubes treated with AAAS and SAAS. Heatmap depicting proteins. The hierarchical clustering results are represented by a tree heatmap. Each column represents a group of samples (the horizontal coordinate is the sample information), and each row represents a protein (the protein with significant difference expression in the vertical coordinate). The expression of the proteins with significant differences in different samples was standardized by the Z Score method and displayed in different colors in the heatmap. Red represents proteins with upregulated significance, blue represents proteins with downregulated significance, and grey represents proteins without quantitative information. **(D)** Upregulation or downregulation in different groups. Volcano plots show different comparisons of the proteomics from the C2C12 myotubes. In the volcano plots, representative signature DEGs were labeled to indicate protein upregulation (red) and downregulation (blue) with the individual treatments when the *p* value was <0.00 s. The x-axis indicates the log^2^ value of the fold change, and the y-axis indicates the-log^10^ of a *p* value. AAAS, medium with asynchronous AA supply; SAAS, medium with synchronous AA supply.

Two myotube samples from the AAAS and SAAS groups were analyzed by LC–MS/MS, and 50,193 unique peptides and 5,004 proteins were identified (Figure 3A). 168 differentially expressed proteins were identified, of which 28 proteins were significantly upregulated and 140 proteins were significantly downregulated (Figure 3B). Heatmap visualization shows the top 100 proteins affected by the asynchronous amino acid supply pattern. Compared with the SAAS group, the asynchronous supply pattern affected three protein clusters associated with protein synthesis and degradation (Figure 3C). Compared with SAAS, the asynchronous AA supply pattern affects the protein synthesis and degradation pathways by downregulating the protein levels of EGFR, PDGFRB, IRS1, and Col5a2 (Figure 3D).

### 3.4 Functional annotation of differentially expressed proteins

**Figure 4:**
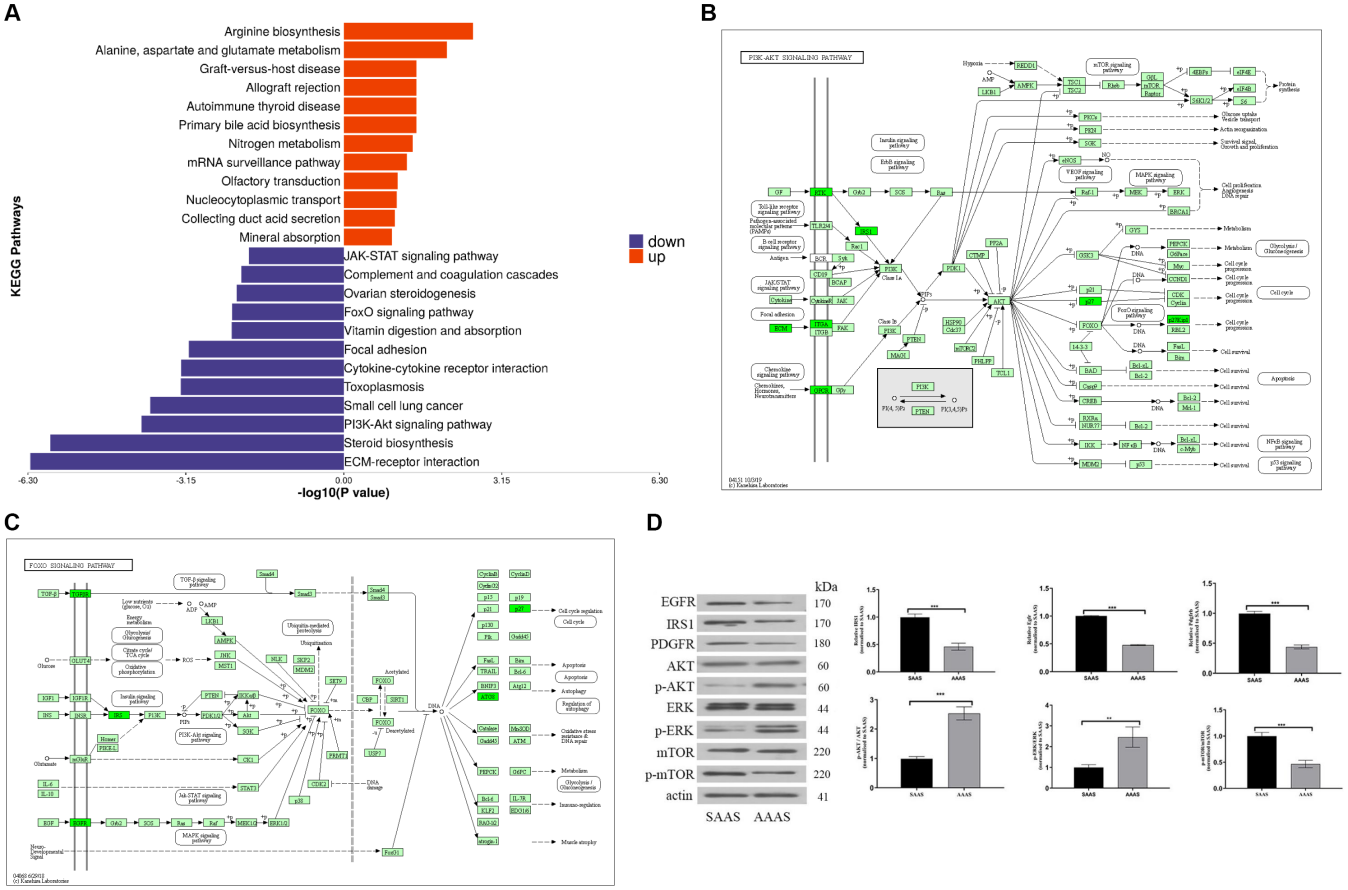
Differential protein pathway enrichment analysis after the AAAS and SAAS treatments. **(A)** Enrichment butterfly diagram of upregulated differentially expressed protein pathways. The horizontal coordinate is the p value of Fisher’s exact test (taking the logarithm of base 10), and the vertical coordinate represents the path name. The pathways involved in upregulated and downregulated proteins are represented by red (right) and blue (left) bars. **(B)** PI3K-Akt signaling pathway diagram of the AAAS vs. SAAS group. **(C)** FoxO signaling pathway diagram of the AAAS vs. SAAS group. Description: The upper left corner is the path name. In the figure, the green bottom box indicates that the proteins with differences are downregulated. The light green bottom box is a species-specific protein. **(D)** Representative immunoblot of EGRF, IRS1, PDGFR, AKT, ERK, mTOR, and phosphorylation of AKT, ERK and mTOR and *β*-Actin. AAAS, medium with asynchronous amino acid supply; SAAS, medium with synchronous amino acid supply.

To better investigate the significance of pathway enrichment for differential proteins, enrichment analysis of KEGG pathways was performed on upregulated and downregulated differentially expressed proteins, which is shown in the form of a butterfly diagram. The results are described as follows (Figure 4A). The upregulated differentially expressed proteins were significantly enriched in the arginine biosynthesis and alanine, aspartate and glutamate metabolism pathways. Downregulated differentially expressed proteins were significantly enriched in ECM-receptor interactions, steroid biosynthesis, the PI3K-Akt signaling pathway, the FoxO signaling pathway and other pathways.

Therefore, we further analyzed the signature EGFR, PDGFRB, and IRS1 differential proteins on the PI3K-Akt/FoxO signaling pathway for protein synthesis and degradation (Figures 4B,C). To verify the expression of the above proteins, Western blot analysis was performed, and the results showed that the AAAS pattern significantly downregulated the expression of IRS1, EGFR and PDGFRB proteins (*p* < 0.05) (Figure 4D). In addition, to study the effects of the AAAS pattern on protein synthesis and degradation of other key proteins, quantitative analysis of Akt and downstream mTOR and their phosphorylated proteins on the PI3K-Akt pathway was performed. The results showed that although the AA supply pattern had no effect on the expression level of mTOR protein, SAAS significantly increased the expression levels of p-Akt and p-mTOR proteins (*p* < 0.05). In addition, we quantitatively analyzed the key protein ERK and its phosphorylated protein on the FoxO signaling pathway. The results also showed that the AA supply pattern had no effect on the expression level of ERK protein, but AAAS significantly increased the expression level of p-ERK protein (*p* < 0.05). Studies have shown that the AAAS pattern regulates protein synthesis and degradation through IRS-1-mediated proteins related to the PI3K-AKT/FoxO signaling pathway.

## 4 Discussion

Digestive kinetics studies have shown that AAs in the diet are asynchronously retained by intestinal and liver tissues (28), leading to an imbalance in amino acids influx into cells, which affects the coordination of AA supply at protein synthesis sites in the body (29). The results were characterized by significant changes in skeletal muscle protein synthesis (30). Therefore, this study reveals protein production and loss during C2C12 myotube synthesis and metabolism by establishing an *in vitro* AAAS pattern.

Skeletal muscle homeostasis is maintained through a balanced relationship between protein synthesis and degradation. We found that desynchronized amino acid release diets affect nitrogen deposition in the body (6, 7), similar to the results obtained in this study; therefore, the AAAS pattern could reduce the efficiency of protein deposition in myotubes, leading to myotube atrophy. This may result from the sensitive response of myoblasts to the regulation of different concentrations of nutrients (31) and because amino acids are the main substrate source for protein synthesis in skeletal muscle; in addition, the balanced concentration of amino acids plays a crucial role in the body’s muscle protein synthesis and metabolism (32). Therefore, when the degradation rate of muscle protein exceeds its synthesis rate, muscle atrophy occurs.

Amino acids are the main reservoir of cellular nitrogen (33, 34). In this study, we revealed the changes in AA in the medium under the AAAS pattern, and the residual glutamate content in the medium of the SAAS group was significantly lower than that of the AAAS group by 6%. This may be because glutamate is used as a nitrogen source during the synthesis of amino acids to maintain the biosynthesis process; however, when not used for biosynthesis, glutamate is converted to free ammonium through the glutamine synthase cycle, and the remaining ammonia is converted to urea (35). Moreover, the asynchronous index of AAAS group was the highest, indicating that the synthesis and metabolism of muscle ducts were in an unbalanced state. It is speculated that this is the reason why AAAS mode reduces protein synthesis and increases urea levels.

The results of this study proved that with the asynchronous supply of amino acids over time, the content of newly formed proteins decreased and the content of urea nitrogen increased, indicating that the synthesis rate of proteins was less than the decomposition rate. Through the enrichment analysis of KEGG pathway, the signal transduction of differential proteins at level 2 mainly belongs to the PI3K-Akt signaling pathway, FoxO signaling pathway, calcium signaling pathway and Rap1 signaling pathway. Previous studies have shown that the PI3K-Akt pathway is a key signal transduction pathway involved in the regulation of cell proliferation, adhesion and differentiation (36, 37). IRS1 is the most important representative of the IRS protein family and a key factor in the PI3K-Akt signaling pathway. During muscle growth, IRS1 is a key medium of PI3K -Akt signal transduction regulating muscle growth and metabolism (38–40). The results of this experiment showed that the expression levels of IRS1, PDGFR and EGFR were significantly reduced by the asynchronous amino acid supply mode during the process of protein synthesis. IRS1 is an important intermediate in the insulin signaling pathway, as well as a key node in the PI3K-AKT pathway, and promotes muscle generation in skeletal muscle (35). EGFR is a transmembrane receptor downstream of IRS1, and its mechanism of action is through ligand-induced dimer initiation signaling cascade (41). It can activate multiple downstream effectors (42), and the downstream transforming growth factor (TGF) can activate EGFR to promote the phosphorylation of signal cascades, and also plays a key role in the communication of PI3K-AKT and other pathways between animal cells (43). Therefore, the non-synchronous supply pattern of amino acids affects the reduction of protein synthesis in muscle ducts, which may be regulated by the IRS1-mediated PI3K-Akt signaling pathway, including protein synthesis, glucose uptake, cell cycle progression and other processes. Therefore, after confirming that the asynchronous pattern of amino acid supply at the protein synthesis site (C2C12 myotube) reduces protein synthesis, it is found that this is achieved by significantly affecting the PI3K-Akt signaling pathway, and by significantly down-regulating the expression levels of EGFR and PDGFRB proteins during the signaling pathway.

In addition, IRS1 protein not only increases protein synthesis by mediating the PI3K pathway, but also blocks the process of protein degradation (44, 45), thereby affecting myotubes hypertrophy (46, 47). Studies have shown that the inhibition of protein degradation is achieved through IRS1-mediated inhibition of Foxo transcription factor family (44, 45). FoxO is activated by upstream signals under conditions of low nutrient supply or starvation. For example, in the liver, a low nutritional state and low levels of insulin signaling activate FoxOs, restoring glucose levels through glycogenolysis and gluconogen (47). Our results also show that the asynchronous amino acid supply pattern significantly affects the expression of IRS1 protein and the activity of FoxO signaling pathway downstream of RTK.

In order to further explore the effects of protein synthesis and degradation on the PI3K-Akt and FoxO signaling pathways, we quantitatively verified the levels and phosphorylation levels of key proteins in the pathways. In mammalian skeletal muscle cells, the expression of Akt protein can lead to muscle hypertrophy (48). Akt is a downstream signal activated by PI3K, and Akt and PI3K together play a role in muscle hypertrophy (49). Therefore, Western blotting is used to detect a certain protein in a sample of sarcotubulin according to the specific binding of the antigen to the antibody. Immunoblotting results showed significant changes in Akt phosphorylation and no changes in Akt. This suggests that the asynchronous amino acid supply mode induces Akt phosphorylation through the PI3K pathway. PI3K-Akt - mammalian target of rapamycin (mTOR) signal transduction is a central pathway controlling cell growth, proliferation and metabolism (50, 51). In this study, mTOR and phosphorylated mTOR were further validated and it was found that the asynchronous amino acid supply mode decreased the level of phosphorylated mTOR protein but did not change the level of mTOR protein. The reason is hypothesized to be due to the activation of Akt, which phosphorylates a range of substrates, including proteins that regulate protein synthesis and gene transcription of cell proliferation. It has been reported that the MAPK pathway plays an important role in the growth of myotubes, and in particular the phosphorylation of ERK is essential for myotube proliferation (52). In this study, the analysis showed that after the asynchronous supply of amino acids, the phosphorylated water of ERK was significantly increased through the reduction of EGFR protein, which promoted the activity of FoxO signaling pathway to regulate protein decomposition. Meanwhile, the results of other key differential proteins were verified to be the same as those of proteome. Therefore, the experiment of myotube-cultured cells demonstrated that the asynchronous effect of dietary amino acid release on nitrogen deposition may be related to the synchronization of amino acid supply at the protein synthesis site (muscle cells) in the body. The molecular mechanism of the effect of the asynchronous amino acid supply pattern on the decrease of protein synthesis is mainly realized through the effect that the synthesis amplitude of PI3K-AKT signaling pathway is smaller than the degradation amplitude of FoxO signaling pathway.

## 5 Conclusion

In summary, *in vitro* experiments confirmed that the asynchronous supply of amino acids reduced the amount of protein deposited in cells. Proteomic analysis identified four distinct proteins and two signaling pathways related to protein synthesis, including the PI3K-AKT signaling pathway and FoxO signaling pathway. By detecting these protein markers, it is possible to accurately evaluate the extent to which protein synthesis is affected. The results of this study have important theoretical significance for further understanding the dynamic requirements of animal protein amino acid nutrition and promoting the precision process of animal nutrition.

## Data Availability

We draw the above figures according to the test data. The datasets analyzed during the current study are available from the corresponding author upon reasonable request. The proteomic results presented in the study are publicly available. These data can be found at: ProteomeXchange Consortium via PRIDE Partner repository with data set identifier PXD051755.

## Footnotes

^1^ http://www.ebi.ac.uk/intact/main.xhtml

^2^ http://string-db.org/
